# AI and IoT-Driven Monitoring and Visualisation for Optimising MSP Operations in Multi-Tenant Networks: A Modular Approach Using Sensor Data Integration

**DOI:** 10.3390/s25196248

**Published:** 2025-10-09

**Authors:** Adeel Rafiq, Muhammad Zeeshan Shakir, David Gray, Julie Inglis, Fraser Ferguson

**Affiliations:** 1 School of Engineering, Computing and Mathematical Sciences, University of Wolverhampton, Wolverhampton WV1 1LY, UK; 2 School of Computing, Engineering and Physical Sciences, University of the West of Scotland (UWS), Paisley PA1 2BE, UK; muhammad.shakir@uws.ac.uk; 3 KubeNet Ltd., Glasgow G51 1PR, UK; david.gray@kubenet.net (D.G.); julie.inglis@kubenet.net (J.I.); fraser.ferguson@kubenet.net (F.F.)

**Keywords:** multi-tenant, network monitoring, decentralisation, machine learning, artificial intelligence

## Abstract

Despite the widespread adoption of network monitoring tools, Managed Service Providers (MSPs), specifically small- and medium-sized enterprises (SMEs), continue to face persistent challenges in achieving predictive, multi-tenant-aware visibility across distributed client networks. Existing monitoring systems lack integrated predictive analytics and edge intelligence. To address this, we propose an AI- and IoT-driven monitoring and visualisation framework that integrates edge IoT nodes (Raspberry Pi Prometheus modules) with machine learning models to enable predictive anomaly detection, proactive alerting, and reduced downtime. This system leverages Prometheus, Grafana, and Mimir for data collection, visualisation, and long-term storage, while incorporating Simple Linear Regression (SLR), K-Means clustering, and Long Short-Term Memory (LSTM) models for anomaly prediction and fault classification. These AI modules are containerised and deployed at the edge or centrally, depending on tenant topology, with predicted risk metrics seamlessly integrated back into Prometheus. A one-month deployment across five MSP clients (500 nodes) demonstrated significant operational benefits, including a 95% reduction in downtime and a 90% reduction in incident resolution time relative to historical baselines. The system ensures secure tenant isolation via VPN tunnels and token-based authentication, while providing GDPR-compliant data handling. Unlike prior monitoring platforms, this work introduces a fully edge-embedded AI inference pipeline, validated through live deployment and operational feedback.

## 1. Introduction

MSPs operate complex, multi-tenant networks under strict service-level agreements (SLAs) that demand rapid fault detection, accurate root-cause diagnosis, and minimal downtime. Conventional monitoring tools such as Prometheus and Grafana provide metrics visibility but lack built-in intelligence for early fault prediction, decentralised inference, and tenant-specific optimisation. The absence of predictive analytics and distributed edge awareness limits the capacity of MSPs to meet real-time service restoration targets across heterogeneous client environments.

MSPs have emerged as key players in the IT sector, offering comprehensive managed services covering IT infrastructure, networking, and security. MSPs such as KubeNet Limited [1] have positioned themselves to provide these services on the customer’s premises, in third-party clouds, or within their proprietary cloud environments, all supported by ongoing assistance and active management. These providers face the growing challenge of optimising multi-tenant networks that span diverse geographic locations, making scalable and decentralised monitoring and visualisation solutions critical.

Traditionally, network monitoring tools like Wireshark [2] have been deployed to provide insights into network performance through packet inspection, traffic flow analysis, and various performance-related metrics. These tools excel not only in graphical data presentation but also in numerical representation [3] across diverse time frames. The fundamental purpose of a network monitoring tool is to ensure the network’s health, providing continuous oversight of network elements and triggering alerts when connectivity issues, system malfunctions, or security vulnerabilities are detected.

In the dynamic and complex realm of large-scale multi-tenant network setups, effective decision-making is no small feat. Network administrators grapple with the challenge of comprehensively understanding network usage and performance to make informed and strategic decisions. To this end, a fundamental requirement emerges: the presentation of network data in a meaningful and actionable manner that empowers administrators to optimise network resource utilisation. Moreover, administrators must remain vigilant, promptly detecting critical network conditions such as excessive resource consumption or security breaches, all while maintaining the privacy and autonomy of each tenant within the shared infrastructure.

A multitude of commercial and open-source network monitoring tools populate the market, yet the development of an industrial-grade AI- and IoT-driven data visualisation tool from scratch remains a formidable and time-consuming endeavour for MSPs managing multi-tenant environments. The open-source community has stepped in to alleviate this burden by offering an array of tools for data visualisation, resource monitoring, and alerting systems, creating a foundational framework for developers [4]. Among these open-source tools, Grafana [5] has emerged as a powerful data visualisation platform, while Prometheus [6] stands out as a time-series data monitoring and alerting tool that can integrate with various sensor data and exporters across multi-tenant networks.

The primary aim of this article is to seamlessly integrate state-of-the-art technologies, such as Prometheus, Grafana, Alert Manager, Grafana Mimir, Grafana Loki, an AI module incorporating machine learning models, Nginx, Docker Swarm, Portainer, and various network metric exporter agents, into a modular architecture that streamlines AI- and IoT-driven monitoring and provides highly available and resilient production-ready infrastructure for multi-tenant networks. The proposed architecture embraces portability by deploying Prometheus on Raspberry Pi devices, turning them into plug-and-play embedded systems within clients’ private networks. This modular and portable approach, combined with a centralised data visualisation and alerting system, heralds a new era of reliability for geographically constrained and complex multi-tenant networks with spatial constraints.

This work advances the state of practice for multi-tenant monitoring by extending the widely adopted Prometheus–Grafana stack with an AI-driven, decentralised architecture. Our contributions are as follows:

- **Comprehensive Modular and Edge-Embedded Monitoring Architecture:** We present a modular architecture leveraging open-source tools such as Prometheus, Grafana, Docker Swarm, and Alert Manager, specifically tailored for multi-tenant MSP environments and the challenges of AI- and IoT-driven monitoring. A novel aspect of this is the edge-embedded monitoring approach, where containerised Prometheus instances are deployed on portable Raspberry Pi devices. These devices act as plug-and-play edge nodes, enabling decentralised and scalable data scraping across geographically distributed client networks.
- **AI-Integrated Predictive Capabilities for Proactive Management:** The system embeds trained machine learning models—including Long-Short-Term Memory (LSTM), K-Means clustering, and Simple Linear Regression (SLR)—directly into the monitoring pipeline. This AI-integrated observability exposes predicted system health, resource usage, and anomaly risk as Prometheus metrics, enabling proactive management and automated alert generation. This integration significantly reduces network downtime and incident resolution time.
- **Operational Validation and Improved Responsiveness:** We highlight the implementation of an intuitive data visualisation and alerting system using Grafana and Alert Manager. The system’s proactive and ML-filtered alerts lead to a significant reduction in false alert notifications, ensuring more focused and effective network management. The system was validated in a one-month production trial across five client networks, demonstrating tangible Service Level Agreement (SLA) improvements and providing reproducible deployment artefacts (configurations, alert rules, and resource measurements) for future adoption.

These contributions collectively provide a reproducible reference architecture for AI-augmented, tenant-aware monitoring in MSP contexts.

The structure of the paper is as follows. Section 2 covers related work, Section 3 outlines the proposed modular architecture with AI- and IoT-driven monitoring capabilities, Section 4 discusses the stack of technology including sensor data integration, Section 5 explains system implementation in multi-tenant networks, Section 6 presents the artificial intelligence models, Section 7 presents the system assessment, and Section 8 concludes the paper.

## 2. Related Work

Recent studies demonstrate the growing interest in integrating AI and IoT technologies for network monitoring and optimisation in distributed environments. In this compilation of research endeavours, authors delve into the creation of innovative systems and methodologies for performance testing, network monitoring, and resource optimisation across diverse computing landscapes.

In the initial study [7], the authors devise a performance testing and network monitoring system tailored for various edge computing clusters. They employ diverse tools and technologies, including Prometheus, node exporter, Docker Containers, Grafana, and Kubernetes. Notably, their system incorporates a deep learning model for object detection, optimising computational distribution to the edge cloud. This aligns with edge computing principles, enhancing resource utilisation by intelligently distributing workloads across the edge cloud infrastructure.

Nicholas Chan, in a complementary article [8], introduces a meticulous workflow that uses the capabilities of the Savio Supercluster. This workflow seamlessly combines historical and contemporary data repositories used by Berkeley Research Computing, culminating in the presentation of multiple use cases through best-fit graphs. The process begins with the meticulous collection of CPU metrics via Telegraf and job-related information from Slurm. The integration of this data into the data visualisation tool Grafana facilitates department-wise usage aggregation. This dynamic approach further offers the opportunity to scrutinise CPU usage at an individual node level, accommodating an array of job profiles and diverse use-case scenarios with remarkable flexibility.

In a separate contribution [9], V. Walter-Tscharf orchestrates a sophisticated monitoring engine with components such as Grafana, Apache Spark v3.0, Graphite, and Docker containers. This comprehensive system is purpose-built to expose vital statistics related to JVMs and network performance. Having successfully developed and deployed this system within a private IaaS cloud, the authors undertake meticulous metric observation and analysis. The infrastructure itself transforms into a programmable platform, facilitating the creation and management of virtual machines. Each node is augmented with Docker, Spark, and CollectD to capture and relay metrics unique to each instance. Subsequently, the data from each server converge into Graphite for in-depth analysis of JVM and network statistics, with Grafana providing a seamless gateway to access a global view of aggregated data spanning the entire cluster.

Sukhija et al. [10] present an architectural blueprint designed to meet the evolving needs of flexible and scalable active network monitoring and data centre management. Their architecture, utilising Grafana, Kubernetes, Prometheus, Node Exporter, and machine learning models for object detection, reflects a forward-thinking approach. Machine learning is skillfully employed to distribute computational workloads across edge nodes, revealing crucial metrics thoughtfully visualised in Grafana. The authors enhance this system with threshold alarms for proactive issue resolution and a comprehensive data visualisation strategy that seamlessly connects data from multiple edge nodes.

Shifting attention to resource monitoring of network elements, Wenyan Chen et al. [11] advocate the use of Prometheus and Perf monitoring tools. Their research focuses on workload measurement and behavioural analysis at the application level, emphasising the role of application workloads in resource allocation and performance optimisation. By scrutinising the characteristics of workloads running concurrently on the same machine, the authors adeptly estimate the interference of each job. Similarly, Y. Huang et al. propose an ingenious edge computing framework tailored to enhance real-time monitoring [12]. To further elevate the performance of real-time monitoring, they introduce a heuristic algorithm founded on a simulated annealing strategy. This algorithm deftly addresses computation scheduling challenges that emerge in the interplay between central and edge cloud components.

This paper presents a novel, AI-enhanced architectural framework for MSPs specifically designed to address the distinct challenges of multi-tenant network management. Unlike previous research, which largely focused on single-tenant monitoring or limited descriptive analytics using tools like Prometheus and Grafana, our work introduces a modular, hybrid edge–cloud architecture that embeds containerised AI models directly within the network environment. This system is tailored to overcome operational constraints unique to MSPs, such as the need for strict tenant isolation, the complexity of heterogeneous device ecosystems, on-site resource limitations, and the challenge of high alert churn and SLA-driven remediation. By integrating IoT sensor data and AI models for predictive fault detection and edge-level decision support within Prometheus nodes, the architecture is positioned to significantly decrease network downtime and incident resolution times for engineers. This not only promises superior service quality and operational efficiency for MSPs and their diverse clients but also furnishes essential production-ready deployment information, representing a significant and novel contribution to the evolving landscape of distributed network management.

## 3. Proposed Modular Architecture

**Figure 1:**
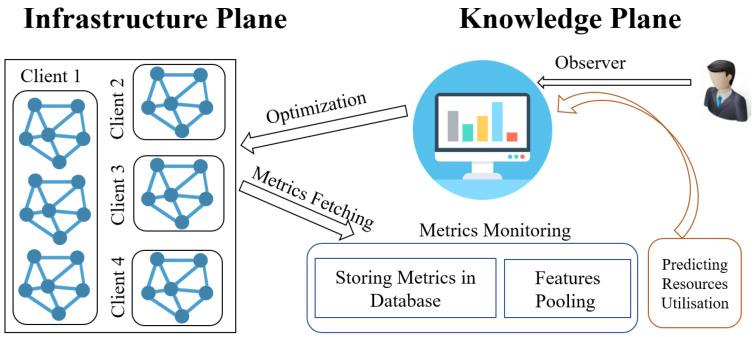
Overall architecture of the proposed AI-integrated monitoring system. The framework comprises two layers: (i) the Knowledge Plane, which consists of metric collection via Prometheus exporters for observation, embedding AI models for predictive analytics, and integration with Grafana and Alertmanager for visualisation and alert routing; and (ii) the Infrastructure Plane, which consists of network devices that generate data and require monitoring and maintenance. Optimisation denotes actions derived from AI predictions, such as early fault alerts and ticket prioritisation, enabling network engineers to optimise network performance after making changes based on these alerts. Directional arrows indicate data and control flows across the components.

In the proposed decentralised AI- and IoT-driven monitoring architecture for multi-tenant networks, influenced by the work of [13], we have outlined two fundamental components as illustrated in Figure 1: the infrastructure plane and the knowledge plane. The infrastructure plane encompasses the multi-tenant networks consisting of geographically distributed client environments. The knowledge plane includes essential elements such as data observation, analysis, alerting systems, AI modules, and Docker Swarm for orchestration. This modular architectural design supports scalable and secure monitoring and visualisation across diverse client networks.

Within the infrastructure plane, a crucial role is fulfilled by data transmission devices that efficiently transfer network and sensor data collected from various IoT devices and network nodes to the knowledge plane for thorough processing and analysis. This approach enables the integration of heterogeneous sensor data from IoT devices into the monitoring system, enhancing the granularity and accuracy of network performance insights.

The knowledge plane acts as the intelligence hub, storing network and sensor data and conducting comprehensive analyses using integrated machine learning models such as SLR, LSTM, and K-Means clustering. These analyses support well-informed decision-making, proactive alerting for anomalies or incidents, and maintain high availability of all core services. The knowledge plane is further enhanced by a data analytics tool facilitating data-driven decisions and alerting based on predefined thresholds. Additionally, a data visualiser provides network administrators with insightful representations, streamlining efforts towards optimising network resources and overall service quality within multi-tenant MSP operations.

The proposed system’s design is inherently tailored to address the monitoring needs of numerous multi-tenant networks, currently encompassing five such networks in its infrastructure plane. These multi-tenant networks consist of diverse network components, including switches, routers, Windows PCs, Windows servers, Linux PCs, GPUs, VMware ESXi machines, MS SQL DB servers, and websites. Importantly, these diverse client networks are dispersed across multiple cities in both Germany and Scotland, exemplifying the complexity and geographic dispersion typical of MSP-managed multi-tenant environments.

In a dependable real-time AI- and IoT-driven monitoring system, establishing a sustainable end-to-end request flow is crucial. The process commences as exporter agents extract metrics and sensor data from the network devices and IoT sensors. Subsequently, Prometheus fetches those metrics from exporter agents and archives these metrics both locally and in Grafana Mimir for prolonged storage. Syslogs from switches undergo a comparable procedure via the Promtail exporter and Rsyslog, being stored in Grafana Loki.

The monitoring tool assesses data from Prometheus, Grafana Mimir, and Grafana Loki, taking predefined threshold-based and AI-informed actions. It presents the information using Grafana’s advanced visualisation tools, enabling network administrators to scrutinise and enhance the behaviour of network devices and IoT infrastructure, thereby improving service quality and operational efficiency as required. The integration of IoT sensor data enriches the system’s ability to detect anomalies and predict potential faults more accurately in the multi-tenant network context.

The system’s proposal underscores its commitment to adhering to established standard criteria for network monitoring systems, ensuring robustness, scalability, and the capability to meet the varied monitoring requirements of our esteemed clients operating complex multi-tenant networks. Moreover, the modular architecture supports the seamless addition of new IoT devices and AI modules, ensuring the system remains adaptable to evolving MSP operational demands.

## 4. Stack of Technologies

**Figure 2:**
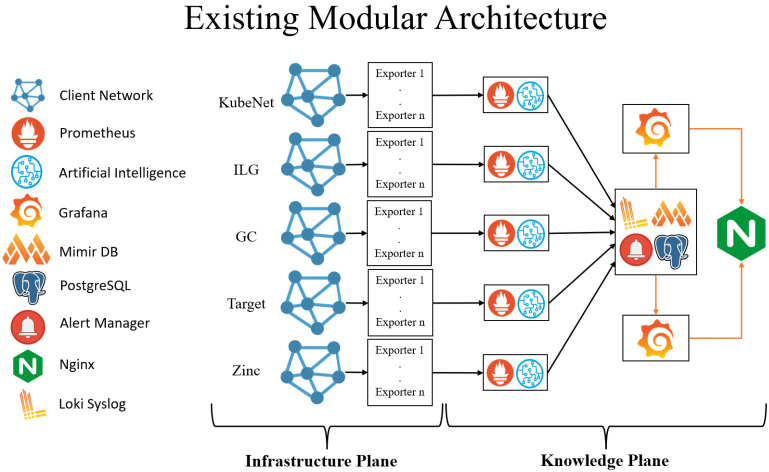
Modular architecture of decentralised monitoring platform. Data flow of the monitoring pipeline work as the metric data is collected from exporters and processed through Prometheus instances hosted on Raspberry Pi edge nodes. Aggregated data is forwarded to the Mimir time-series database for long-term storage and AI model training. Trained models are containerised and deployed either centrally or at the edge for inference. Predictions are exposed as Prometheus metrics and visualised in Grafana dashboards. This architecture enables hybrid edge–cloud analytics, real-time alerting, and scalable tenant isolation.

The entire suggested framework adopts a modular architecture designed specifically for AI- and IoT-driven monitoring and visualisation of multi-tenant networks within MSPs. As illustrated in Figure 2, each module functions autonomously within its own restricted private network, supporting scalable and tailored monitoring environments for diverse clients. To implement this modular, scalable, and secure platform, we rely on a comprehensive collection of open-source tools, each contributing to the real-time, proactive network monitoring and performance optimisation objectives central to MSP operations.

Training is performed centrally on anonymised data using a GPU-enabled server, after which model artefacts are containerised and versioned. For inference, two options are supported: (1) edge deployment on Raspberry Pi, exposing predicted risk scores as Prometheus metrics; or (2) central inference, where predictions are published back to Mimir for aggregation. The prediction endpoint uses the native Prometheus alerting rules can be found here [14].

### 4.1. Exporters Agents

In this IoT-enabled monitoring platform, exporters serve as vital components by collecting metrics from various network nodes and transforming raw sensor data into formats digestible by Prometheus. Prometheus utilises an HTTP pull model to extract metrics from these exporters, enabling continuous real-time data collection [15]. These exporters cover a broad spectrum of network elements, including node-exporter for Linux devices, windows-exporter for Windows systems, VMware-exporter for ESXi hosts, MSSQL-exporter for SQL servers, SNMP-exporter for routers and switches, NVIDIA GPU exporter for GPU monitoring, and logging exporters such as Promtail and Rsyslog feeding into Grafana Loki for comprehensive log aggregation. BlackBox-exporter further enhances the monitoring scope by performing active network probing. These exporters effectively act as IoT sensor data collectors, seamlessly integrating heterogeneous network components into the AI- and IoT-driven platform, enabling MSPs to visualise network health and performance accurately.

While many exporters collect virtual telemetry (e.g., SNMP, node metrics), Raspberry Pi Prometheus units operate as edge IoT nodes, functioning as embedded gateways aggregating both physical and logical sensor data. Future extensions will integrate true environmental sensors (e.g., power, temperature, humidity).

### 4.2. Prometheus

At the core of the modular architecture, Prometheus functions as the principal real-time monitoring and data storage system for each client, embodying the AI- and IoT-driven vision of the platform. It facilitates efficient data collection, storage, and alerting across multi-tenant environments. Prometheus’s Time-Series Database (TSDB) stores critical network and IoT metrics based on predefined KPIs. Despite its limited native visualisation capabilities, Prometheus’s strong querying functionality underpins the platform’s ability to provide rich, actionable insights. Integration with Grafana enhances the visualisation and alerting capabilities, empowering MSP operators with intuitive dashboards and timely notifications crucial for optimising service quality in complex, geographically dispersed networks.

### 4.3. AI Models

The AI-driven component of the system leverages advanced machine learning techniques, including Simple Linear Regression (SLR), Long Short-Term Memory (LSTM) neural networks, and K-Means clustering, to transform collected IoT sensor data and monitoring metrics into predictive insights. The LSTM model predicts future network states, enabling proactive detection of potential system faults an hour in advance. K-Means clustering categorises network node health status into distinct states—healthy, warning, or fault—facilitating real-time condition monitoring. SLR forecasts resource utilisation trends such as memory and disk usage, providing early warning signals. By embedding these AI models directly within the monitoring platform, the system empowers MSPs to automate incident detection, minimise downtime, and improve operational efficiency across multi-tenant networks.

### 4.4. Grafana Mimir

Grafana Mimir extends Prometheus’s capabilities by providing scalable, long-term storage and global querying functionality, essential for managing the vast amounts of IoT and network data generated across MSP multi-tenant environments [16]. Supporting up to 1 billion active time series, Grafana Mimir enhances the platform’s ability to maintain persistent, reliable data archives, allowing for deep historical analysis and improved decision-making. Its high-availability architecture guarantees data durability and supports the AI- and IoT-driven platform’s scalability and resilience needs.

### 4.5. Grafana Loki

Grafana Loki serves as the log aggregation engine within the modular system, designed to efficiently store and query logs generated by various network components and IoT devices [17]. Through integration with Promtail, Loki collects logs from dispersed sources, providing MSPs with rich contextual information vital for troubleshooting and forensic analysis. Loki’s scalable, multi-tenant architecture fits perfectly within the AI- and IoT-driven platform, enhancing visibility into network behaviour and supporting faster root cause identification.

### 4.6. Alert Manager

Alert Manager orchestrates the alerting mechanisms for the platform, leveraging Prometheus’s rule-based query system [18]. It consolidates and manages alerts from multiple services, enabling fine-grained routing, grouping, and de-duplication. With support for complex routing rules, templates, and silence periods, Alert Manager ensures timely and accurate notifications to MSP service desk operators and network architects. This AI- and IoT-driven alerting process reduces alert noise and empowers proactive responses, thereby improving service quality and network uptime.

### 4.7. Grafana

As the platform’s front-end, Grafana delivers a powerful and user-friendly interface for visualising real-time and historical data, supporting MSP operators in making informed decisions. Its rich ecosystem of plugins and support for multiple data sources make it ideal for representing complex multi-tenant network metrics gathered through IoT sensor integrations. Customisable dashboards and interactive features allow users to drill down into specific network segments or client environments, enhancing situational awareness and enabling data-driven optimisation of MSP operations.

### 4.8. Nginx

Nginx acts as a robust load balancer and reverse proxy within the system, optimising access to Grafana instances and enhancing the overall platform’s availability and security [19]. Proper configuration of domain names and proxy settings ensures seamless user experience when interacting with dashboards, especially in a multi-tenant MSP context. Nginx supports high availability by routing traffic across multiple Grafana instances, contributing to the resilience of the monitoring and visualisation platform.

### 4.9. Docker Swarm

Docker Swarm is the orchestration engine deployed to manage and scale the modular system’s containerised services [20]. It supports multi-node clustering, ensuring that services are highly available and resilient to node failures—a critical requirement for MSP environments managing multiple tenant networks. This container orchestration enables rapid deployment, updates, and scaling of AI, Prometheus, Grafana, and supporting modules, underpinning the system’s ability to adapt to changing resource demands and client requirements.

### 4.10. Portainer

Portainer provides an intuitive management UI for Docker Swarm clusters, simplifying the operational oversight of containerised components [21]. Through its comprehensive management of containers, images, networks, and volumes, Portainer enhances the usability and maintainability of the modular architecture. It assists MSP administrators in effectively managing multi-tenant deployments, ensuring smooth system operation and rapid troubleshooting.

This comprehensive stack of technologies—combining open-source tools with AI and IoT integration—empowers Managed Service Providers to deploy a scalable, resilient, and intelligent platform for real-time monitoring and visualisation of complex multi-tenant network infrastructures. By bridging IoT sensor data acquisition, AI-driven predictive analytics, and advanced data visualisation, the system delivers actionable insights that optimise MSP operations, elevate service quality, and enable proactive network management in increasingly intricate environments.

## 5. Putting the System into Practice

This section provides practical insights into the implementation of the modular architecture for AI- and IoT-driven real-time network monitoring and visualisation, as discussed earlier. In line with Figure 2, each client corresponds to a dedicated Prometheus instance, a necessity imposed by private network security restrictions limiting public exposure. To overcome this challenge, a portable, modular monitoring solution was developed, utilising Raspberry Pi boards as compact IoT nodes hosting Prometheus instances, ensuring isolated and secure monitoring within individual client networks [22]. The remaining core components of the proposed system operate on three virtual machines deployed in the cloud, hosted within KubeNet’s infrastructure to support scalable multi-tenant network monitoring across geographically distributed environments.

**Figure 3:**
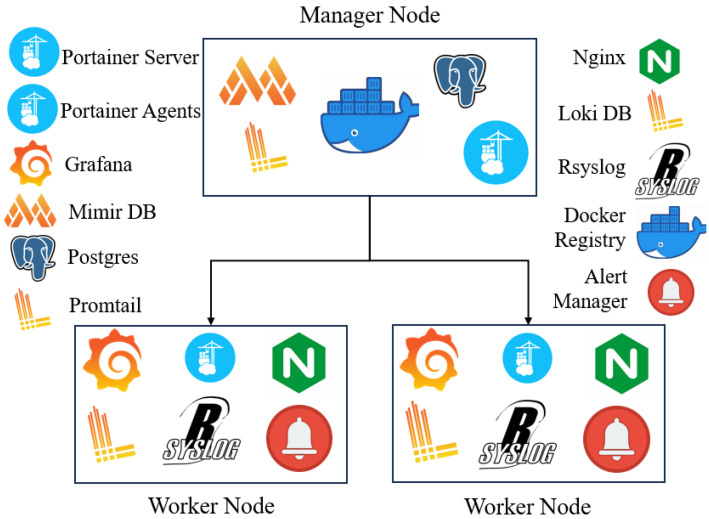
Production-ready, highly available, and resilient deployment using Docker Swarm.

The architectural diagram (Figure 3) illustrates a Docker Swarm cluster designed for a production-ready, multi-service, multi-node deployment, embodying a resilient and highly available system. This orchestration platform integrates 11 distinct services that collectively constitute the network monitoring and visualisation ecosystem. The Docker Swarm manager node ensures continuous operation of all critical services, rapidly recovering any failed service within seconds to maintain uninterrupted monitoring capabilities. If a worker node goes offline, other workers uphold full service availability, reflecting the system’s designed resilience and high availability—essential for MSPs managing diverse client networks with minimal downtime.

Within this deployment, five separate Prometheus instances run on Raspberry Pi boards acting as edge IoT devices, collectively overseeing nearly 500 network elements distributed across five client organisations. This decentralised monitoring approach leverages multiple data exporters integrated with Prometheus, capturing rich sensor data streams from the multi-tenant network infrastructures.

The complete deployment comprises three nodes: one manager and two worker nodes. Each node hosts specific services carefully allocated based on resource needs and operational roles. The Manager Node runs PostgreSQL for persistent storage of Grafana dashboards and configurations, and Grafana Mimir, providing permanent long-term storage for time-series data vital for AI-driven predictive analysis. Grafana Loki, tasked with log management, also resides on the manager node, ensuring permanent storage of syslogs and network event data. The Docker Registry service is hosted on this node, securely storing container images for system components. Finally, a Portainer server provides an intuitive UI for cluster visualisation and management, simplifying operations for MSP administrators.

Worker Nodes host two Grafana instances deployed across separate nodes, each integrated with Grafana Mimir to enable comprehensive data visualisation tailored to client-specific multi-tenant requirements. Additionally, two Loki Promtail instances collect and forward logs to Grafana Loki, while two Alert Manager instances operate redundantly for robust alert delivery. All Prometheus servers send alerts to Alert Manager, triggering timely notifications. Enhancing security and access performance, two Nginx proxy instances balance and secure connections to Grafana. Two Rsyslog instances on worker nodes aggregate system logs, interfacing closely with Loki Promtail. Lastly, two Portainer agents facilitate Docker API request proxying and response aggregation across the cluster.

This portable real-time AI- and IoT-driven network monitoring system integrates seamlessly into client networks, pairing Prometheus metrics collection with Grafana’s powerful data visualisation, Grafana Mimir’s long-term data retention, and Alert Manager’s automated email notification capabilities targeted at service desk teams and network design engineers. To safeguard device identities and restrict public exposure, a Virtual Private Network (VPN) exclusively connects the Raspberry Pi Prometheus instances to Grafana and Alert Manager. Currently, evaluation is underway to extend VPN protection for communications between Prometheus and Grafana, further securing sensitive multi-tenant data exchanges from external threats. Importantly, the portable monitoring system benefits from localised communication between metrics exporters and Prometheus within the same private network, eliminating the need for complex VPN configurations, firewall rule modifications, or port forwarding, thus enhancing security and simplifying deployment.

Prior to deployment within client networks, each Raspberry Pi module undergoes comprehensive preparation tailored to client-specific configurations. The selected hardware platform consists of Raspberry Pi 4 units with 512 GB SD cards, running Ubuntu 22.04 LTS and equipped with Ethernet for robust network connectivity. Each Prometheus instance is paired with a dedicated group of network metrics exporters and containerised AI modules, ensuring granular data acquisition and intelligent analytics for each client’s multi-tenant environment. Table 1 details the specific exporters deployed either as standalone services or in conjunction with Prometheus on Raspberry Pi units across clients.

On the Raspberry Pi edge, we used 512 GB high-endurance SD cards for short term retention (local retention = 90 d). Prometheus WAL settings were as follows: wal-compression: true; wal-segment-size: 64 MB. Remote_write to Mimir is configured with a write queue to buffer during WAN outages (max_shards: 1, min_backoff: 30 ms, max_backoff: 5 s). For production we recommend external SSD or industrial eMMC; the revised manuscript discusses trade-offs and provides a conversion table comparing write endurance.

The monitoring system is designed to supervise approximately 500 network nodes in total, with Table 1 highlighting a subset of data exporters used. Exporter selection is driven by adaptability and coverage: some exporters operate as services within Linux and Windows systems, interfacing with Prometheus through northbound or southbound APIs. VMware ESXi constraints necessitate Docker containerised exporters paired with Prometheus on Raspberry Pi setups, enabling southbound API communication with ESXi hosts and northbound integration with Prometheus.

During the trial we observed approximately 350,000 active time-series (measured with the Prometheus series API), with common scrape intervals of 60 s depending on exporter. The Mimir cluster was configured with RF = 3 and horizontal sharding as described; 95th percentile query latency during peak was less than 200 ms.

In environments where third-party software installation is restricted—such as Cisco and HP network devices—exporters run within Raspberry Pi containers, communicating via southbound APIs to routers and switches, while forwarding metrics to Prometheus through northbound APIs. Similarly, MSSQL exporters operate within Docker containers on Raspberry Pi, adapting to client restrictions and ensuring seamless data flow for database performance monitoring. Other exporters deployed within Raspberry Pi environments facilitate monitoring of HTTP services, SSL certificate expiry dates, and network node connectivity using ICMP protocols. The system also utilises Rsyslog integrated with Prometheus on Raspberry Pi setups, where network nodes forward syslog data to Rsyslog, which then formats and transmits logs to Grafana Loki for long-term storage.

Alert Manager instances efficiently manage email alert notifications, with each Prometheus instance configured individually to route alerts. Two redundant Alert Manager instances ensure high availability and reliability in delivering alerts to MSP operational teams. The architecture’s two Grafana instances connect to Grafana Mimir for data visualisation and Grafana Loki for log visualisation. To maintain strict organisational separation, distinct entities with dedicated databases are created within each Grafana instance for every client, effectively providing each organisation with an independent visualisation environment. VPN-based network connections securely link Raspberry Pi Prometheus nodes at client sites with Grafana and Alert Manager, completing the decentralised, secure multi-tenant monitoring system.

This practical deployment demonstrates how the AI- and IoT-driven modular architecture, combined with open-source tooling and container orchestration, facilitates robust, scalable, and secure real-time network monitoring and visualisation tailored to the complex operational demands of Managed Service Providers.

### 5.1. Methodology

This section summarises the end-to-end pipeline from data collection to model validation.

1. **Data acquisition:** Metrics were gathered via Prometheus exporters (node, SNMP, MSSQL, GPU, blackbox) deployed on client sites. Each Raspberry Pi collected and forwarded samples of CPU, memory, storage, and service reachability metrics, etc.
2. **Preprocessing:** Data were standardised, outliers removed, and records sampled every 30th row to align with a one-hour prediction horizon. Ambiguous mid-range values (65–75%, 85–95%) were excluded to reduce label noise. After applying the filters, approximately 1.5 million rows were removed from the clustering pipeline.
3. **Label creation:** Healthy/Warning/Fault states were generated by threshold-based rules validated against service-desk tickets. Labels were defined as follows: Fault if (CPU > 95% OR Memory > 95% OR Storage > 95%), *Warning* if (50% < CPU ≤ 95% OR Memory 50–95%), and Healthy otherwise. Where service-desk tickets existed for the same time window, labels were cross-validated and corrected manually.
4. **Model training:** K-Means, SLR, and LSTM models were trained centrally using Python v3.9 and TensorFlow v2.16.1. Cross-validation followed a 70/15/15 temporal split per host.
5. **Deployment:** Trained models were containerised using Docker and deployed either on Raspberry Pi devices for edge inference or centrally for aggregation.
6. **Validation:** Performance metrics included accuracy, RMSE, precision, recall, and macro-F1. Operational feedback was collected from engineers across five client sites.

Model training was performed on an on-premise machine (Intel Core i9, 64 GB RAM, RTX 3090 GPU). LSTM training required approximately 3.2 h of wall time (batch_size = 64, epochs = 50, early stopping enabled). The saved LSTM model footprint was approximately 12 MB. For edge inference, the container was deployed to a Raspberry Pi4. The median inference latency was measured at ∼140 ms per sample, and peak CPU usage during inference was below 45%, with sufficient thermal headroom (no throttling events recorded).

### 5.2. Security and Compliance

Tenant isolation is ensured through dedicated organisations within Grafana and tenant identifiers within Mimir. Each tenant’s data is strictly partitioned, with dashboards and access tokens scoped per organisation. Exporters communicate locally within client LANs, while Prometheus–Mimir traffic is secured using TLS with mutual authentication (mTLS) and bearer tokens.

Grafana enforces role-based access control (RBAC) via OIDC-based single sign-on (SSO), ensuring user authentication and authorisation are centrally managed. Secrets are handled securely using Docker Swarm secrets during the prototype phase, with Vault recommended for production environments.

GDPR and Retention: All monitoring data is pseudonymised, excluding personal identifiers. A Data Protection Impact Assessment (DPIA) has been completed, confirming compliance with data minimisation and lawful processing under the legitimate interest basis for service quality improvement. Local data retention is limited to 90 days and remote retention to 365 days. Upon contract termination or data deletion requests, records are removed using Mimir’s tenant deletion API, ensuring complete lifecycle management.

## 6. Artificial Intelligence Models

The selected algorithms balance interpretability, deployment simplicity, and resource constraints on edge devices. K-Means offers intuitive unsupervised grouping for system health states; LSTM captures temporal dependencies in sequential telemetry; SLR serves as a lightweight baseline for linear resource trend estimation. Although these models are mature, their integration into a live MSP observability stack with edge inference constitutes a novel systems contribution.

In this section, we provide detailed explanations of the artificial intelligence (AI) models integrated within our AI- and IoT-driven monitoring and visualisation platform designed to optimise MSP operations in complex multi-tenant network environments. These AI models play a pivotal role in enhancing the quality of service through proactive network health prediction and anomaly detection. The dataset used for training and evaluation comprises approximately 9 million rows and 7 columns (timestamp, Memory, Storage, CPU, Bandwidth, Processes, and Exceptions), expressed as percentages except for Processes and Exceptions, collected over a one-year period from more than 50 Windows and Linux systems. The data is time-series in nature, labelled for supervised learning where applicable, and ordered sequentially for each system, making it well-suited for unsupervised machine learning and neural network-based predictive models. The AI component’s objective is not to develop novel machine learning algorithms but to strategically employ existing models that maintain the system’s simplicity while significantly improving MSP operational efficiency.

The granularity and diversity of system states depend on both the complexity of the monitored environment and the categorisation scheme used for classifying network health and system behaviour. In the context of our AI- and IoT-driven monitoring system for Windows and Linux servers, we focus on key performance metrics such as CPU, Memory, Disk Storage, Bandwidth, Processes, and Exception events to categorise system states into meaningful health statuses, enabling MSP teams to make timely, data-driven decisions:

- **Healthy:** The system is operating within normal parameters, with no significant issues affecting performance or stability.
- **Warning:** The system’s performance is noticeably impacted, likely due to high resource usage, bottlenecks, or minor faults.
- **Critical Fault:** The system experiences severe issues that affect its functionality or stability, such as crashes, major errors, or resource exhaustion.

### 6.1. K-Means Clustering

Clustering analysis plays a crucial role in unveiling concealed patterns and structures within extensive datasets, facilitating data-driven decision-making across diverse fields. In this study, we explore the architecture and methodology underpinning an advanced clustering model tailored for efficient analysis of client data. Our approach follows a multi-step process, commencing with meticulous data pre-processing and filtering to ensure the quality of input for subsequent analysis. The dataset used for clustering analysis undergoes initial labelling removal. After loading the data, the filtering operation is executed to eliminate specific rows from the dataset. This filtering is governed by predefined conditions associated with columns such as CPU, Memory, and Storage, targeting metrics containing metric value ranges (percentage buckets of resource utilisation) between 65% to 75% and 85% to 95%. Any rows meeting these conditions are excluded from further analysis. To promote uniformity and bolster clustering accuracy, we leverage the StandardScaler algorithm to standardise feature scales.

**Figure 4:**
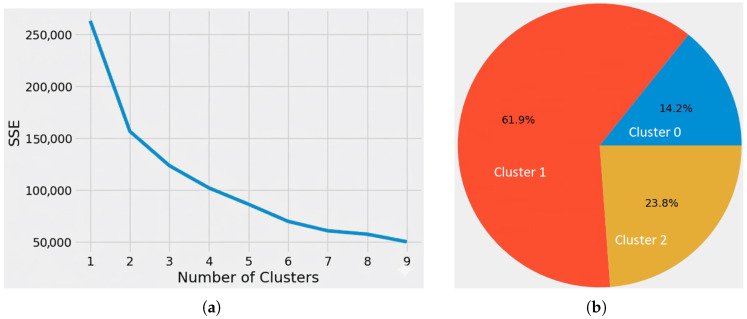
K-Means Clustering performance analysis. (**a**). Optimal solution for finding the number of clusters in the dataset. (**b**). Pie-chart showing the distribution of cluster lengths.

The heart of our model lies in the determination of the optimal number of clusters, a critical yet challenging task in clustering analysis. Leveraging the K-means algorithm, we iterate through a range of potential cluster counts, evaluating the Sum of Squared Errors (SSE) to identify the elbow point, indicative of the optimal clustering solution. To automate this process, we utilise the KneeLocator algorithm, streamlining the selection of the ideal cluster count based on the SSE vs. Number of Clusters plot as shown in Figure 4a.

Because the SSE curve did not show a pronounced elbow, we evaluated a range of k using silhouette and Davies–Bouldin indices and inspected cluster centroids for operational interpretability. K = 3 was selected because it provided a robust silhouette score and produced centroids that map directly to Healthy, Warning, Fault states used by operators (centroid CPU/memory/storage thresholds).

The resulting clusters from K-means are interpreted by analysing the cluster centroids (or means) as the primary criteria, specifically looking at the average value for each feature (cpu, memory, disk, etc.) within that cluster which is reported in Table 2. Interpretation involves comparing these mean values across all clusters to identify which features are unusually high or low for a particular group. For instance, a cluster with a high average cpu and disk usage, but a low exception rate, would be profiled as “CPU-Bound Workstations,” allowing the statistical profile to be translated into a meaningful, actionable business label.

Once the optimal cluster count is identified, our model reverts to the K-means algorithm, employing the specified number of clusters to partition the dataset effectively. This iterative process ensures robust and insightful clustering outcomes, facilitating enhanced data interpretation and decision-making capabilities as shown in Figure 4b.

Figure 4b illustrates the number of clusters corresponding to the states of the systems described in the preceding subsection. In this context, cluster 1 signifies the healthy state of a system, cluster 2 denotes the warning state, and cluster 0 indicates the critical fault state of the system.

To illustrate the optimization function for K-Means clustering:

(1) $$J=\sum_{i=1}^{k}\sum_{x\in C_{i}}{∥x-\mu_{i}∥}^{2}$$

where *J* is the total within-cluster sum of squares (objective function), *k* is the number of clusters, $C_{i}$ is the set of points in cluster *i*, *x* represents the data point, and $\mu_{i}$ is the centroid of cluster *i*.

### 6.2. Long Short-Term Memory (LSTM)

Long Short-Term Memory (LSTM) neural networks have emerged as a powerful tool for modelling sequential data, particularly in tasks such as time series classification. In this section, we elaborate on the LSTM architecture employed in our research to classify time series data related to system behaviour.

The architecture begins with the pre-processing of the dataset containing time series data. Subsequently, the dataset undergoes reduction by selecting every 30th row, followed by normalisation of features using the StandardScaler library. This normalisation step is crucial for ensuring that each feature contributes proportionally to the model’s learning process, thereby preventing biases due to differences in feature scales.

Upon pre-processing, the dataset is transformed into sequences of data points, each representing a window of observations with a specific sequence length. These sequences are constructed with a one-hour prediction target, enabling the model to learn temporal dependencies and make predictions about future system behaviour. The construction of sequences is a pivotal step in preparing the data for training the LSTM model.

The LSTM architecture itself comprises multiple layers stacked sequentially. The first layer is an LSTM layer with 64 units, followed by a dropout layer with a dropout rate of 0.2 to prevent over-fitting. Subsequently, another LSTM layer with 32 units is added to capture additional temporal patterns in the data. The output layer consists of a dense layer with softmax activation, facilitating multi-class classification by predicting the probabilities of each class.

During model compilation, the Adam optimiser is utilised along with the sparse categorical cross-entropy loss function, while accuracy serves as the evaluation metric. Additionally, early stopping and model checkpointing techniques are incorporated as callbacks to monitor the model’s performance during training and save the best-performing model based on validation loss.

**Figure 5:**
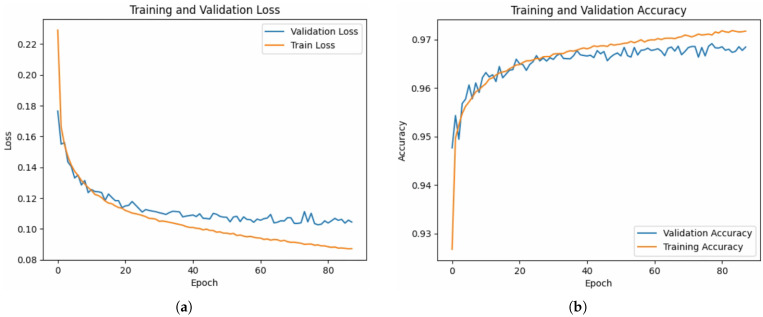
LSTM performance analysis. (**a**). Line graph showing training and validation loss. (**b**). Line graph showing training and validation accuracy.

The trained model’s performance is assessed by analysing the training and validation loss, as well as the training and validation accuracy, over epochs as depicted in Figure 5. This assessment provides insights into the model’s capability to learn from the data and generalise to unseen instances. Figure 5a illustrates the training and validation loss, while Figure 5b displays that LSTM has achieved 97.1% classification accuracy on the test set, with per-class metrics reported in Table 3.

**Figure 6:**
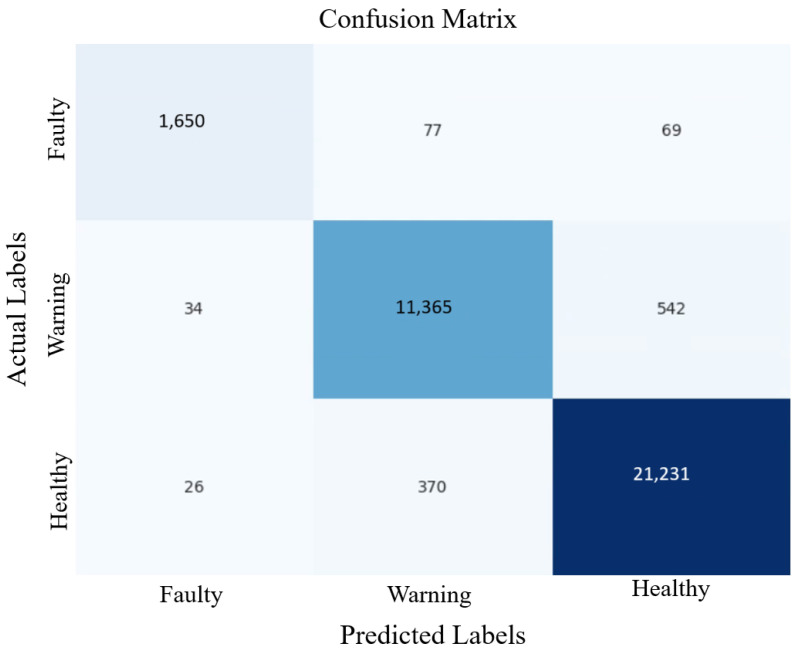
Confusion matrix for validating the confidence of the LSTM model.

Precision, recall, and macro-F1 scores confirm robustness across all states. The 2 min raw sampling rate was down-sampled by selecting every 30th record, producing one-hour intervals consistent with the target prediction horizon. Additionally, the model’s classification accuracy is evaluated through the generation and analysis of a confusion matrix, as presented in Figure 6, providing a comprehensive understanding of its effectiveness in classifying system behaviour.

To detail the computations of the LSTM cell:

(2) $$f_{t}=\sigma \left(W_{f}\cdot \left[h_{t-1},x_{t}\right]+b_{f}\right)$$

(3) $$i_{t}=\sigma \left(W_{i}\cdot \left[h_{t-1},x_{t}\right]+b_{i}\right)$$

(4) $${\tilde{C}}_{t}=\tanh\left(W_{C}\cdot \left[h_{t-1},x_{t}\right]+b_{C}\right)$$

(5) $$C_{t}=f_{t}*C_{t-1}+i_{t}*{\tilde{C}}_{t}$$

(6) $$o_{t}=\sigma \left(W_{o}\cdot \left[h_{t-1},x_{t}\right]+b_{o}\right)$$

(7) $$h_{t}=o_{t}*\tanh\left(C_{t}\right)$$

where $f_{t}$, $i_{t}$, and $o_{t}$ are the forget, input, and output gates, respectively. $W_{f}$, $W_{i}$, $W_{C}$, and $W_{o}$ are the weight matrices, and $b_{f}$, $b_{i}$, $b_{C}$, and $b_{o}$ are the bias terms. $C_{t}$ represents the cell state at time *t*, $h_{t}$ is the hidden state at time *t*, and $x_{t}$ is the input at time *t*.

### 6.3. Simple Linear Regression (SLR)

**Figure 7:**
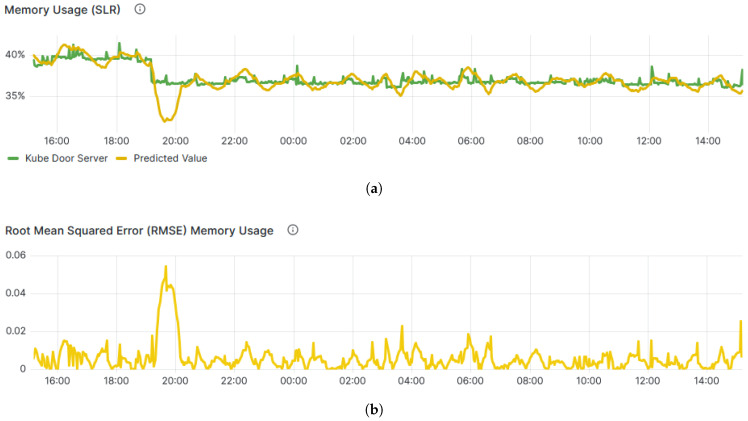
SLR performance analysis. (**a**). Line graph showing actual and predicted memory usage. (**b**). Line graph showing the RMSE values in predicting memory usage.

In the proposed system, SLR is employed to forecast resource utilisation, a crucial aspect of the Prometheus server. In SLR, we use a short window of lagged values (range vector) as the predictor set; the independent variable X refers to elapsed time or an aggregated index within that window, used for simple short-term linear trend forecasting. Figure 7a demonstrates the real and predicted values of Memory usage over the past 1 h, showcasing the level of confidence in prediction. Moreover, the precision of the SLR model, assessed through Root Mean Square Error (RMSE) and illustrated in Figure 7b, holds significant importance in ensuring accurate alert notifications, effectively indicating the attainment of predefined thresholds.

SLR is used as a simple short-horizon baseline model; its predictions are interpreted with caution and compared against LSTM performance using RMSE and classification metrics.

To demonstrate how SLR is used to predict resource utilisation, the following equation is used:

(8) $$Y=\beta_{0}+\beta_{1}X+\epsilon$$

where *Y* is the predicted resource utilization (e.g., memory usage), *X* is the independent variable (e.g., time or other metrics), $\beta_{0}$ is the intercept, $\beta_{1}$ is the slope of the line (coefficient), and ϵ is the error term.

On the other hand, RMSE can be calculated as follows:

(9) $$\mathrm{RMSE}=\sqrt{\frac{1}{n}\sum_{i=1}^{n}{\left(y_{i}-{\hat{y}}_{i}\right)}^{2}}$$

where *n* is the number of observations, $y_{i}$ is the actual value, and ${\hat{y}}_{i}$ is the predicted value.

The integration of K-Means clustering, LSTM neural networks, and SLR within the AI- and IoT-driven monitoring and visualisation platform significantly enhances the ability of MSPs to detect, predict, and respond to network performance issues proactively. By employing these AI techniques alongside IoT-generated sensor data, the platform delivers sophisticated, real-time insights that improve operational efficiency and service quality across complex multi-tenant network environments.

## 7. System Performance Assessment

Baseline performance values were derived from KubeNet operational logs covering the six months preceding deployment. Downtime denotes cumulative unavailability (in minutes) per node (up == 0), while resolution time measures the interval between alert generation and service restoration as recorded in support tickets. The sample size per client was 100 tickets. As the mean time between failures (MTBF) increased significantly, the number of tickets requiring resolution decreased drastically. Comparisons were conducted using identical service teams to ensure consistency.

The proposed AI- and IoT-driven monitoring and visualisation platform was evaluated to assess its effectiveness in optimising MSP operations within complex multi-tenant network environments. The evaluation primarily focused on improving the response time of service desk and network design engineers who manage customer complaints and troubleshoot network issues. By leveraging the modular architecture and AI-enhanced monitoring capabilities, the system facilitates quicker diagnosis of problems and significantly reduces network downtime.

**Figure 8:**
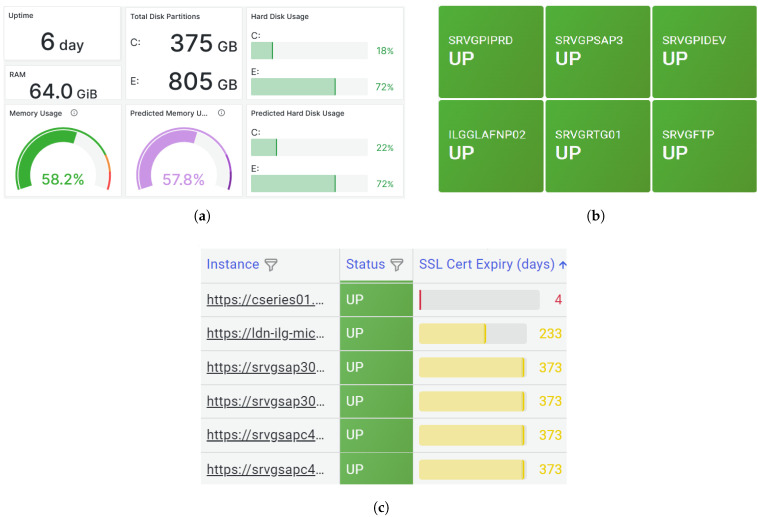
Snapshot of panels from the multiple dashboards. (**a**). Windows system with predicted metrics. (**b**). Current status of system connectivity. (**c**). Current status of websites.

**Figure 9:**
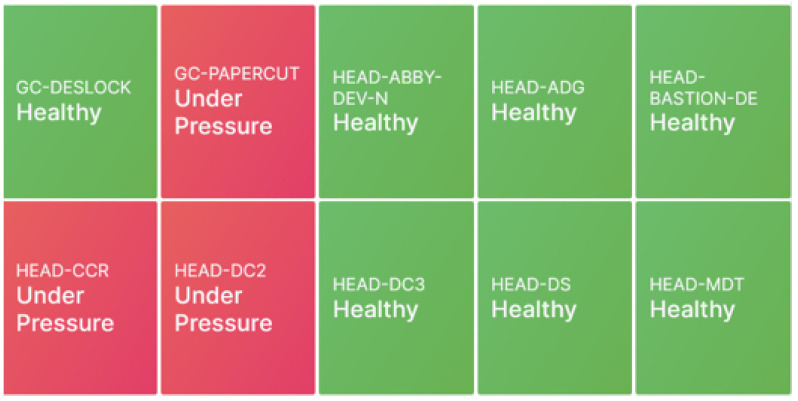
Snapshot from the dashboard showing the predicted system health status. Where green indicates a healthy network state, orange signals a warning, and red highlights critical faults.

Figure 8 and Figure 9 illustrate key functionalities of the developed system. Figure 9 presents the system health assessment through the K-Means clustering model, where green indicates a healthy network state, orange signals a warning, and red highlights critical faults. In Figure 8a, the dashboard provides a comprehensive overview from a Windows machine, displaying metrics such as RAM, hard disk usage, and CPU utilisation. Figure 8b shows the real-time connectivity status of network-connected machines, with visual indicators transitioning from green (connected) to red (disconnected). Figure 8c demonstrates monitoring of website accessibility and SSL certificate expiry, enabling proactive certificate renewals. These functionalities are underpinned by the use of ICMP for network connectivity checks and HTTP protocols for website and SSL monitoring. This comprehensive and visually intuitive data presentation enhances situational awareness for MSP service operators and network architects alike.

Beyond visualisation, the system incorporates automated alert criteria configured within each Prometheus instance. These alerts generate email notifications upon detection of five consecutive failed ICMP or HTTP responses, enabling rapid intervention by network teams to prevent client impact. Another critical alert triggers notifications seven days prior to SSL certificate expiry, supporting proactive maintenance. Additionally, Simple Linear Regression (SLR) models predict resource utilisation thresholds, sending alerts when anticipated usage surpasses predefined limits. Similarly, LSTM-based predictions of overall network node behaviour contribute to alert generation, enhancing the timeliness and relevance of notifications. This AI-driven alerting mechanism reduces the weekly alert noise from an overwhelming 70–75 notifications to a manageable 10–14, by filtering out 80–85% of false positives, thereby allowing the MSP teams to focus on genuine issues.

**Figure 10:**
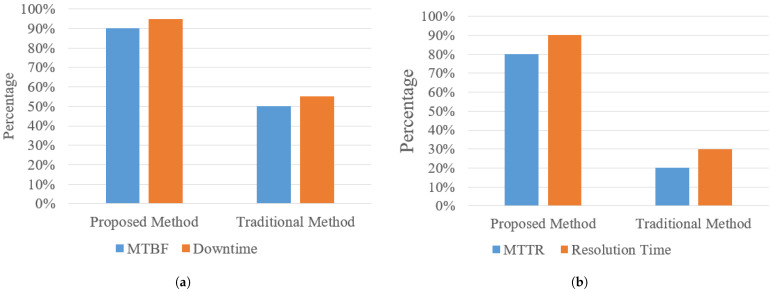
Percentage improvement in (**a**) total downtime and (**b**) incident resolution time relative to historical baseline. Values represent relative change computed as (baseline−trial)/baseline×100. Error bars indicate 95% confidence intervals across five MSP clients.

As discussed in Section 2, the unique challenges of multi-tenant network monitoring have not been sufficiently addressed by existing solutions. Therefore, a comprehensive real-world case study was conducted over one month across five distinct clients, collecting 54 pieces of valuable feedback from service desk and network design engineers. The results, depicted in Figure 10a,b, demonstrate a remarkable 95% reduction in network downtime and a 90% decrease in incident resolution time compared to traditional manual methods.

Traditionally, manual monitoring and incident handling have achieved up to a 50% reduction in downtime and mean time between failures (MTBF). The proposed system surpasses these benchmarks, significantly extending network reliability and uptime. Similarly, improvements in mean time to repair (MTTR) and resolution time with manual approaches typically reach 45%, whereas the AI- and IoT-driven platform accelerates resolution efforts dramatically, as reflected in the empirical data. These improvements highlight the platform’s transformative impact on operational efficiency, user experience, and overall network availability.

A practical example from user feedback illustrates this transformation: prior to the system’s deployment, issue reporting through user tickets or calls typically took 5 to 10 min, followed by an additional 5 to 10 min for the service desk to respond. With the new platform continuously monitoring client sites, immediate email alerts enable the team to begin diagnostics before users even report problems. In many cases, the issue can be resolved within the initial 5 to 10 min of detection or even faster, often requiring only a simple system restart taking less than five minutes, compared to the previous 20 to 45-min resolution window. This enhanced responsiveness yields nearly one hour saved per alert, translating directly into increased network uptime and improved client satisfaction.

In compliance with stringent company security policies and General Data Protection Regulation (GDPR) agreements, data displayed in this study has been carefully limited and anonymised. Nonetheless, KubeNet’s quarterly reports confirm high client satisfaction levels and consistent reductions in system downtime, underscoring the value of integrating AI, IoT, and data visualisation technologies within MSP operations. The system’s scalability and modular design further ensure that as client networks evolve and grow, the monitoring platform can adapt seamlessly to maintain optimal service quality.

## 8. Conclusions

This research has presented a comprehensive AI- and IoT-driven monitoring and visualisation platform specifically designed to optimise MSP operations within multi-tenant network infrastructures. The proposed system leverages a modular architecture that integrates sensor data from diverse IoT devices with advanced machine learning techniques, including Simple Linear Regression (SLR), Long Short-Term Memory (LSTM) networks, and K-Means clustering, to deliver proactive monitoring, early incident detection, predictive analytics, and automated decision-making. By utilising open-source technologies such as Prometheus for high-performance data collection and Grafana for intuitive visualisation, the platform ensures transparency, scalability, and adaptability in diverse MSP contexts.

The findings from experimental evaluations demonstrate that the platform can achieve up to a 95% reduction in network element downtime and a 90% decrease in incident resolution time. These improvements directly enhance service quality, operational efficiency, and client satisfaction key performance indicators for MSP organisations managing complex multi-tenant network environments. Furthermore, the scalable modular design ensures that the system can be tailored to different MSP operational needs, accommodating network growth, emerging IoT device integrations, and evolving AI capabilities.

This study demonstrates a practical architecture that integrates AI-driven predictive analytics within an open-source monitoring stack for MSP operations. The modular design enables portability across client sites and supports hybrid inference at the edge and cloud. Operational evaluation confirms substantial gains in service reliability and responsiveness.

Future work will focus on two fronts: (i) Incorporating reinforcement learning to dynamically tune alert thresholds and remediation policies, optimising between sensitivity and false positives; (ii) Extending the pipeline to include cybersecurity anomaly detection using IDS and firewall logs, broadening applicability to threat-aware network monitoring.

In conclusion, the proposed AI- and IoT-driven monitoring and visualisation platform offers a novel, scalable, and impactful approach to optimising MSP operations. By uniting cutting-edge AI techniques, IoT sensor networks, and interactive visual analytics, the system establishes a foundation for smarter, faster, and more resilient network management in the era of complex, data-rich, and interconnected service environments.

## Figures and Tables

**Table 1:** Varieties and quantity of data exporters for each client.

Exporter	Client-1	Client-2	Client-3	Client-4	Client-5
Windows	5	1	7	20	26
Blackbox	1	3	1	1	1
MSSQL	1	3	4	2	1
SNMP	1	1	1	1	1
Rsyslog	1	1	1	1	1
GPU	1	0	0	0	0
Linux	1	0	0	0	0
VMware	1	0	0	0	0

**Table 2:** Interpretation of resulting K-means clusters based on centroid analysis.

Cluster	Defining Characteristics (Criteria)	Interpretation/Label
Cluster 0	Low cpu (∼20.15), Low memory (∼21.74), Low exception (∼0.54)	**Low Utilisation / Idle Systems:** This cluster represents systems that are generally running smoothly with minimal resource demand.
Cluster 1	Very High cpu (∼99.91), High disk (∼81.28), Low rw_latency (∼11.01)	**CPU-Bound / Heavy Computational Load:** The near 100% average CPU utilisation clearly defines this group. These systems are under severe computational stress.
Cluster 2	High memory (∼76.70), High c_drive (∼78.05), Highest exception (∼1.90)	**Memory/Storage/Error Prone:** The highest average memory usage and C-drive utilisation, coupled with the highest exception count, suggests these systems may be struggling with memory management or application errors.

**Table 3:** Performance metrics for LSTM state classification.

Class	Precision	Recall	F1	
Healthy	0.96	0.98	0.97	
Warning	0.95	0.94	0.94	
Fault	0.97	0.95	0.96	
Macro Avg	0.96	0.96	0.96	–

## Data Availability

The data supporting the findings of this study contain sensitive information derived from a production environment. Therefore, they cannot be publicly shared. However, access to the cleaned and anonymised dataset may be granted upon reasonable request and subject to institutional approval.
